# Lack of HIV infection among truck drivers in Iran using rapid HIV test

**Published:** 2010

**Authors:** Hossain Jabbari, Seyed Ahmad Seyed Alinaghi, Parastoo Kheirandish, Gholam Reza Esmaeli Djavid, Mehrnaz Rasolinejad, Mahboube Hajiabdolbaghi, Abbas Sedaghat, Maryam Sargolzaei, Amir Houshang Sharifi, Minoo Mohraz, Kaveh Khoshnoud

**Affiliations:** a Iranian Research Center for HIV/AIDS (IRCHA), Tehran University of Medical Sciences, Tehran, Iran; b Academic Center for Education, Culture and Research (ACECR), TUMS Branch, Tehran, Iran; c Ministry of Health and Medical Education, Tehran, Iran; d Department of Epidemiology and Public Health, Yale University School of Medicine, New Haven, USA

**Keywords:** Human Immunodeficiency Virus, Truck, Automobile Driving

## Abstract

**BACKGROUND:**

The aim of this study was to evaluate the prevalence of HIV infection in Iranian long distance truck drivers using rapid HIV test.

**METHODS:**

The study included 400 consecutive participants in Bazargan city, north-west of Iran in the late 2008 and the early 2009.

**RESULTS:**

No HIV infection was observed among these long distance truck drivers.

**CONCLUSIONS:**

Although results of this study is plausible compared to other similar studies, repeated surveys are necessary to know the trend of HIV infection in truckers in Iran.

Several studies have shown that long distance truck drivers (LDTDs) who spend most of their time traveling in different cities far from their family and sexual partners are at high risk of HIV infection through high risk sexual contacts with sex workers and have been used as a sentinel population in surveillance programs.^1^–^3^ In a study in Brazil, HIV prevalence among Brazilian truck drivers mentioned to be about 1.3%,^4^ while in several studies in Kenya and Uganda their prevalence was 25% to 32%.^5^

To our knowledge, no similar study has been conducted among this population in Iran. This study assessed the prevalence of HIV infection among LDTDs as a sentinel population for HIV/AIDS using the rapid HIV test.

## Methods

In this study, 413 consecutive LDTDs (all men) were approached from November 2008 to January 2009. Of these, 400 participants consented to attend anonymously. This study was approved by institutional review board (IRB) of Tehran University of Medical Sciences (TUMS) and was done in Bazargan city located in north-west of Iran, at the border of Iran and Turkey. The truckers were traveling to Middle East and European countries and they were approached while waiting at the border. Basic demographic information (age and marital status) was recorded in data collection sheet. After obtaining verbal consent, 40 micro liter of blood was collected by sampler for rapid HIV test (KHB, Inc., Shanghai, China). The test results were read after 3-5 minutes.

## Results

Among the participated truckers, the mean age was 36 years (range: 22-64 years), and 324 (81%) were married. No HIV positive test was found among this sample of Iranian LDTDs in the Iran-Turkey border.

## Discussion

In this study, no HIV positive test was found. Possible reasons for this result include: 1) there is truly very little HIV infection in Iranian LDTDS, ad 2) this sample represents lower risk LDTDS compared to samples of LDTDS in other countries. Although in this study positive test results was not found, other studies have shown some positive results (Table 1). A major limitation of this study is that no question was asked about risk behaviors among these participants. Despite this limitation, the current study provides the first estimate of HIV seroprevalence among LDTDs in Iran. Regarding similar studies in other countries (Table 1), periodical tests in this sentinel population along with necessary training in order to improve insight, knowledge and preventive measures about HIV/AIDS appear to be essential. Future studies are suggested to evaluate behavioral risk factors in truck drivers regarding paucity of data in this population in Iran. Finally, expanding similar studies at other borders which may attend more high risk LDTDs is suggested too.

**Table 1:** Prevalence of HIV infection among truck drivers in other studies

Country	Sample size	Prevalence
East Africa^6^	970	27%
Kenya^5^	283	26%
India^2^	2066	4.6%
Brazil^4^	300	1.3%
Tongling, China^7^	550	0%

## Conclusions

Our data shows that HIV infection prevalence in LDTDS, as a low risk sentinel population, performed based on WHO protocols is less than 1%. This rate is lower than most other similar subpopulations studied. Repeatedly such surveys help to sketch better picture of HIV infection trend in Iranian truckers.
